# Polymerase θ inhibition steps on the cGAS pedal

**DOI:** 10.1172/JCI170660

**Published:** 2023-06-01

**Authors:** Chelsea M. Smith, Gaorav P. Gupta

**Affiliations:** 1 Lineberger Comprehensive Cancer Center,; 2 Department of Pathology and Laboratory Medicine, and; 3 Department of Radiation Oncology, University of North Carolina, Chapel Hill, North Carolina, USA.

## Abstract

Deficiencies in homologous recombination (HR) repair lead to an accumulation of DNA damage and can predispose individuals to cancer. Polymerase theta (Pol θ, encoded by *POLQ*) is overexpressed by HR-deficient cancers and promotes cancer cell survival by mediating error-prone double-stranded break (DSB) repair and facilitating resistance against poly-ADP ribose polymerase inhibitor treatment. In this issue of the *JCI*, Oh, Wang, et al. report on the impact of Pol θ inhibition on activation of antitumor immunity. The authors used pancreatic ductal adenocarcinoma (PDAC) cell and mouse models characterized by HR-associated gene alterations and *POLQ* overexpression. *POLQ* knockdown showed synthetic lethality in combination with gene mutations involving DNA repair, including *BRCA1*, *BRCA2*, and *ATM*. Notably, Pol θ deficiency or inhibition suppressed tumor growth, increased the accumulation of unrepaired DNA damage, and enhanced T cell infiltration via the cGAS/STING pathway. These findings suggest a broader scope for Pol θ inhibition in HR-deficient cancers.

## HR-deficient cancers rely on Pol θ for viability

Our cells are constantly exposed to both internal and external stressors that damage DNA. The most lethal type of DNA lesion is a double-strand break (DSB), which, if not repaired, can result in cell death, or, if misrepaired, can result in carcinogenic mutations. The homologous recombination (HR) pathway accurately repairs these DNA breaks. HR functions in S and G2 phases of the cell cycle, where sister chromatids can serve as templates for DNA repair. Deficiencies in HR repair can lead to cancer, and mutations in HR genes (e.g., *BRCA1*, *BRCA2*, and *PALB2)* are linked to hereditary breast and ovarian cancers. Loss of HR function leads to a characteristic accumulation of chromosomal rearrangements, including deletions and templated insertions that often harbor short tracts of sequence identity at the breakpoint junctions (i.e., microhomology) (1).

We now recognize that this pattern of microhomology-associated rearrangements induced in HR-deficient cells is due to the upregulation of an alternative DSB repair pathway mediated by DNA polymerase theta (Pol θ, encoded by *POLQ*) (2–4). Theta-mediated end joining (TMEJ) accounts for the majority of mammalian microhomology-mediated end joining (MMEJ) and entails initial resection of the DSB end (similar to HR) followed by template-independent end joining through microhomology-driven hybridization and synthesis (5, 6). Pol θ has a unique ability to prime (and reprime) synthesis with as little as one nucleotide of microhomology, which can give rise to complex mixtures of deletions and templated insertions that appear to be TMEJ specific (7, 8). Remarkably, this error-prone DSB pathway is evolutionarily conserved across plant and metazoan species, presumably due to an essential role in preventing certain types of catastrophic DNA damage (9).

HR-deficient cancers exhibit *POLQ* overexpression and are highly dependent on Pol θ for their viability (10, 11). Particularly, since most healthy cells seem to tolerate Pol θ deficiency, the synthetic lethality between *POLQ* inactivation and HR deficiency has inspired efforts to discover and develop Pol θ inhibitors as cancer therapeutics. Two Pol θ inhibitors are already being investigated in clinical trials (NCT04991480, NCT05687110), with several more anticipated soon. There is hope that Pol θ inhibitors will help overcome primary and acquired resistance to Poly ADP-Ribose Polymerase (PARP) inhibitors, which is an urgent challenge in the clinical treatment of HR-deficient cancers (12, 13). However, the optimal strategies to deploy Pol θ inhibitors for maximal therapeutic benefit remain unclear and require further investigation.

## Pol θ inhibition activates the immune system

**Figure 1:**
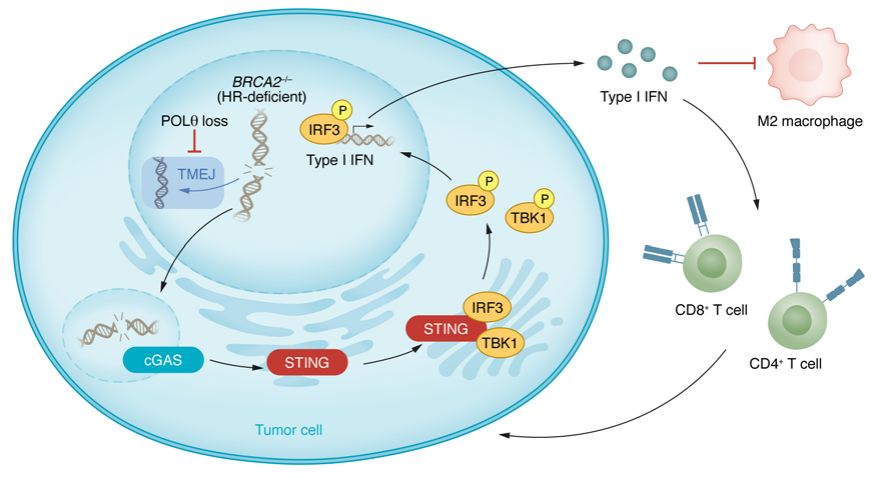
Pol θ inhibition stimulates cGAS/STING to promote T cell-directed anti-tumor immunity. *BRCA2^-/-^* PDAC tumor cells compensate for the loss of HR by upregulating Pol θ, which mediates TMEJ and is essential for repairing DSBs. Inhibition of Pol θ results in accumulation of cGAS-positive micronuclei and STING activation. The cGAS/STING pathway activates TBK1 and IRF3 via phosphorylation, initiating Type I IFN production and release. Subsequent increases in CD4^+^ and CD8^+^ T cell infiltration with decreasing immunosuppressive M2-like TAMs result in suppression of tumor growth.

In the current issue of the *JCI*, Oh and Wang, et al. report findings that reveal the impact of Pol θ inhibition on immune activation (Figure 1) (14). Their study focuses on pancreatic ductal adenocarcinoma (PDAC), motivated by the observations that nearly a quarter of PDAC cases are associated with HR-associated gene alterations and that *POLQ* overexpression correlates with worse prognosis in two independent cohorts of PDAC patients. Pol θ was a synthetically lethal target in patient-derived and murine PDAC models in combination with mutations in either of the HR genes *BRCA1* or *BRCA2*, or in *ATM*, which is an upstream kinase that responds to DSBs and activates signaling events required for HR repair. Genetic knockdown of *POLQ* and treatment with Pol θ inhibitors resulted in broad growth inhibition and accumulation of unrepaired DNA damage in HR-deficient PDAC models, relative to PDAC models that lacked HR-associated gene mutations. Furthermore, the authors observed additive — and possibly synergistic — effects when combining Pol θ inhibitors with PARP inhibitors in HR-deficient models. These observations validate Pol θ as a promising therapeutic target in HR-deficient PDAC (14).

Oh, Wang, and authors went on to demonstrate that Pol θ inhibition in HR-deficient (i.e., *BRCA1*, *BRCA2*, and *ATM* mutant) PDAC stimulated the accumulation of cyclic GMP-AMP synthase–positive (cGAS-positive) micronuclei. cGAS and its downstream partner stimulator of IFN genes (STING) constitute a cytosolic DNA sensing pathway that activates the innate immune system through transcriptional activation of IFN-stimulated genes (14, 15). While the innate immune system primarily serves as a defense against exogenous DNA during viral or bacterial infection, it also senses damaged DNA within the cell’s cytosol. Hence, the cGAS/STING pathway functions in tumor surveillance and in the immunological response to cancer therapy by responding to an accumulation of unrepaired DNA.

The authors also showed that Pol θ inhibition followed by cGAS/STING activation had therapeutically relevant immunological consequences. Through in vivo analysis of a *Brca2*-deficient murine PDAC model, the authors demonstrated that Pol θ inhibition increased intratumoral CD4^+^ and CD8^+^ T cell infiltration, while tumor-associated macrophages (TAMs) decreased, primarily due to a decrease in immunosuppressive M2-like TAMs. These effects were dependent on STING, as knockdown of STING diminished these effects, despite Pol θ targeting, resulting in partial restoration of tumor growth (14). These results are consistent with a recently published complementary study by Patterson-Fortin et al., which demonstrated that Pol θ inhibition increased micronuclei formation, cGAS/STING pathway activation, and CD8^+^ T cell influx (16). Additionally, the Patterson-Fortin et al. study observed an upregulation of PD-L1 expression and saw additive effects of Pol θ inhibition with anti-PD-1 therapy in HR-deficient triple–negative breast cancer and PDAC models (16).

## Unresolved questions and translational implications

These two recent studies demonstrate that, in certain HR-deficient settings, Pol θ inhibition has a substantial impact on immune activation through stimulation of the cGAS/STING pathway (14, 16). The precise genetic contexts wherein this process may occur remains to be further clarified. While *BRCA2*-deficient tumors seem to be most consistently sensitive to Pol θ inhibition, it’s not currently clear whether all *BRCA1*-deficient tumors will be Pol θ dependent, and even less is known about tumors with mutations in other synthetically lethal gene targets such as *PALB2*, *ATM*, or others (17). While Oh and Wang, et al. provided evidence that Pol θ inhibition in *ATM*-mutant PDAC stimulates cGAS/STING activity, in vivo tumor growth effects were not evaluated (14).

Another important and unresolved question is whether all Pol θ inhibitors will exhibit similar biological effects. Both studies used the same two Pol θ inhibitors in their experiments (14, 16). ART558 is a recently identified allosteric Pol θ inhibitor (13, 18). In contrast, Novobiocin is an antibiotic with known activity against a bacterial DNA gyrase that was shown to also inhibit ATPase activity of the Pol θ helicase–like domain (12, 19). While Patterson-Fortin et al. (16) primarily investigated Novobiocin in their studies, Oh, Wang, et al. (14) found greater HR-deficient tumor selectivity with ART558. An improved understanding of these Pol θ inhibitors, including the functional implications of their distinct selectivity profiles and mechanisms of action, will be needed for optimal clinical translation.

Another unresolved question is: how do the present findings relate to a prior observation that PARP inhibition can also induce cGAS/STING-dependent immune responses in HR-deficient cancers (20)? Does all unrepaired DNA damage stimulate equivalent levels of cGAS/STING activation, or could there be synergistic effects? Emerging data indicate that Pol θ may have unique roles for DNA repair in mitosis, which raises the possibility that Pol θ may provide an important last defense against micronucleus formation and subsequent innate immune activation (21–23). Thus, the potential treatment of combined PARP and Pol θ inhibition may be warranted to maximally synergize with immune checkpoint inhibitors in HR-deficient cancers.

## Outlook

The studies by Oh and Wang, et al. (14) and Patterson-Fortin et al. (16) create new directions for exploring the clinical impact of Pol θ inhibitors through combination with immunotherapy. More work is needed to clarify the tumor genetic contexts that are most susceptible to immune stimulation by Pol θ inhibition and reveal the optimal biomarker to identify such cases clinically. Additionally, a deeper mechanistic understanding of how mitotic DNA repair by Pol θ protects against immune activation may yield therapeutic synergies with relevance beyond HR-deficient cancers.

## Version 1 06/01/2023

Electronic publication
